# Assessment of Lipoprotein(a) in Patients with Cancer: Relevance for Cardio-Oncology Practice

**DOI:** 10.3390/ijms272110055

**Published:** 2026-11-09

**Authors:** Małgorzata Wójtowicz, Wiktor Bortnowski, Alicja Grzenkowicz, Zuzanna Lewicka-Potocka, Dorota Smolarek, Grzegorz Sławiński, Ludmiła Daniłowicz-Szymanowicz, Ewa Lewicka

**Affiliations:** 1 Students’ Scientific Circle at the Department of Cardiology and Electrotherapy, Medical University of Gdańsk, 80-214 Gdansk, Poland; 2 Cardio-Oncology Outpatient Clinic, University Clinical Center, 80-214 Gdansk, Poland; 3 Department of Cardiac Diagnostics, Medical University of Gdańsk, 80-214 Gdansk, Poland; 4 Department of Cardiology and Electrotherapy, Faculty of Medicine, Medical University of Gdańsk, 80-214 Gdansk, Poland

**Keywords:** lipoprotein(a), cardio-oncology, cancer, atherosclerotic cardiovascular disease, cardiovascular risk assessment, cancer therapy-related cardiovascular toxicity, biomarker

## Abstract

Cardiovascular disease is a major cause of morbidity and mortality in patients with cancer and in cancer survivors. Lipoprotein(a) [Lp(a)] is a largely genetically determined causal risk factor for atherosclerotic cardiovascular disease and calcific aortic valve disease, acting through proatherogenic, proinflammatory, and potentially prothrombotic pathways. These mechanisms may be relevant in cancer, where inflammation, hypercoagulability, metabolic disturbances, and treatment-related cardiovascular toxicity increase cardiovascular risk. Observational studies have also linked circulating Lp(a) to tumor characteristics, disease stage, or prognosis in several malignancies. However, these findings are heterogeneous, often derived from small, retrospective, or single-center cohorts, and do not establish a causal role for Lp(a) in cancer development or progression. Evidence relating Lp(a) specifically to cancer therapy-related cardiovascular toxicity remains limited, and a prospective biomarker cohort found no association between baseline Lp(a) and major adverse cardiovascular events. This review summarizes the biology and measurement of Lp(a), critically evaluates its reported oncological associations, and examines its relevance in cardio-oncology. Currently, Lp(a) should be interpreted primarily as part of comprehensive cardiovascular risk assessment rather than as a cancer-specific or cardiotoxicity biomarker. Prospective, treatment-specific studies are needed to determine whether Lp(a) improves risk prediction or represents an actionable therapeutic target in this population.

## 1. Introduction

Advances in cancer detection and treatment have substantially improved survival, but have also increased the number of patients exposed to short- and long-term cardiovascular complications. Cardiovascular risk in patients with cancer results from the complex interplay among pre-existing cardiovascular disease, conventional cardiovascular risk factors, cancer-associated inflammation and thrombosis, treatment-related metabolic disturbances, and the direct and indirect cardiovascular toxicity of anticancer therapies [1,2,3]. Improved risk stratification is therefore a central objective of cardio-oncology.

Lipoprotein(a) [Lp(a)] is a common, predominantly inherited lipoprotein trait and an established causal risk factor for atherosclerotic cardiovascular disease (ASCVD) and calcific aortic valve stenosis [4,5,6,7,8,9,10,11]. Approximately one in five individuals has an Lp(a) concentration exceeding the commonly used threshold for increased ASCVD risk; nevertheless, Lp(a) measurement remains underutilized in routine clinical practice [12]. Because Lp(a) concentrations are largely genetically determined and generally stable throughout adulthood, measuring Lp(a) at least once in every adult’s lifetime should be considered. The result should be incorporated into the assessment of lifelong cardiovascular risk [13,14]. This principle is relevant to patients with cancer, whose baseline risk may be amplified by anticancer therapy and competing clinical priorities.

Interest in Lp(a) in oncology extends beyond conventional cardiovascular prevention. Observational studies have reported associations between Lp(a) and the occurrence, phenotype, stage, or prognosis of several cancers. The direction of association is not consistent across tumor types, and circulating concentrations can be altered by hepatic dysfunction, inflammation, hormonal status, nutritional state, and anticancer treatment. Consequently, Lp(a) may represent an inherited cardiovascular risk factor, a marker of tumor- or host-related biology, or both. These interpretations must not be conflated.

This narrative review examines three distinct questions: (1) how Lp(a) should be measured and interpreted in patients with cancer; (2) whether Lp(a) is associated with cancer biology or outcomes; and (3) whether Lp(a) improves prediction or management of cancer therapy-related cardiovascular toxicity (CTR-CVT). The distinctive contribution of this review is its focus on clinical significance of Lp(a) in cardio-oncology exceeding previously described connections with cancer prevalence and prognosis. Particular emphasis is placed on the strength and limitations of the available evidence.

The literature was searched using a non-systematic, targeted approach, in line with the narrative nature of this review. Topic-specific searches were conducted in PubMed using combinations of the following terms: “lipoprotein(a)”, “Lp(a)”, “cancer”, “cardio-oncology”, specific cancer types (breast, lung, prostate, ovarian, colorectal, gastric, pancreatic, and hepatocellular cancer), “atherosclerotic cardiovascular disease”, “cardiovascular risk assessment”, “cancer therapy-related cardiovascular toxicity”, and “biomarkers”. No lower date limit was set, and the last search was conducted on 15 September 2026. Studies were selected based on their importance to the review objectives, such as Lp(a) and cancer incidence, prognosis, treatment response, and cardiovascular toxicity.

## 2. Biology and Measurement of Lipoprotein(a)

### 2.1. Structure, Genetics, Biological and Molecular Effects

Lp(a), first described by Kåre Berg in 1963, comprises an LDL-like particle containing apolipoprotein B-100 (apoB-100) covalently linked to apolipoprotein(a) [apo(a)] [5]. The LPA gene determines the number of kringle IV type 2 repeats in apo(a). This copy-number variation produces marked apo(a) size polymorphism and, in general, an inverse relationship between apo(a) isoform size and circulating Lp(a) concentration [5,15]. Concentrations are predominantly genetically determined, become established early in life, and remain relatively stable in most adults.

Lp(a) contributes to vascular disease through several overlapping pathways. Its LDL-like moiety promotes cholesterol deposition, whereas Lp(a) is also a major carrier of oxidized phospholipids that stimulate endothelial activation, monocyte recruitment, and vascular inflammation [6,7]. Because apo(a) is structurally homologous to plasminogen, Lp(a) may interfere with fibrinolytic pathways, although the clinical importance of this mechanism varies by context [15,16]. This interaction reduces plasmin generation and slows clot degradation, thereby promoting thrombosis. The mechanism involves the kringle V domain and the lysine-binding site in KIV-10, rather than the inactive protease-like domain [17]. Recent observational data suggest that the association between Lp(a) and venous thromboembolism may be modified by sex and hormonal exposure, supporting a context-dependent rather than universal thrombotic effect [18,19,20].

**Figure 1:**
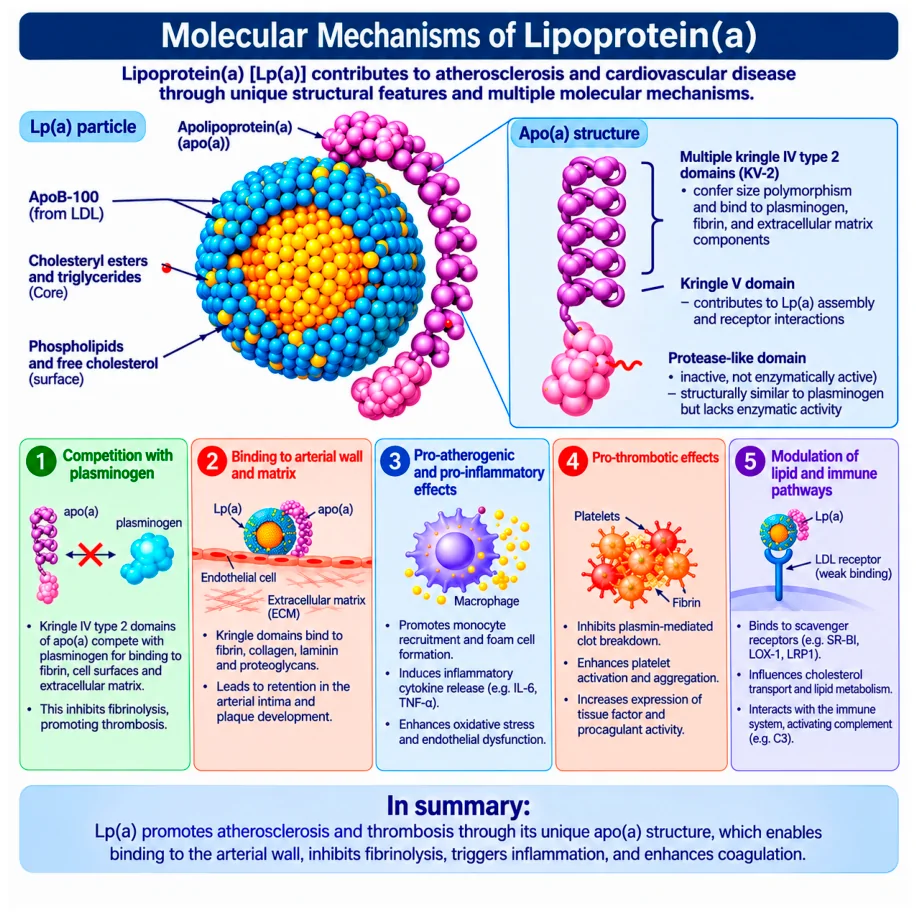
Molecular mechanisms of lipoprotein(a) [Lp(a)] contributing to atherosclerosis and thrombosis. The main pathophysiological mechanisms are presented, including competition with plasminogen and inhibition of fibrinolysis (Hancock et al., 2003 [17]), binding to the arterial wall and extracellular matrix, promotion of pro-atherogenic and pro-inflammatory responses, enhancement of oxidative stress and endothelial dysfunction (Boffa and Koschinsky, 2019 [24]), platelet activation and pro-thrombotic effects (Rand et al., 1998 [21]), and modulation of lipid metabolism and immune pathways, including interactions with the complement system (Seifert et al., 1992 [25]). Source: Created by the authors using OpenAI’s GPT-6 model and ImageGen image-generation tool, based on published scientific literature. The generated figure was reviewed and scientifically verified by the authors.

The causal association between elevated Lp(a) and ASCVD is supported by genetic, epidemiological, and mechanistic evidence [5,8,15]. Current data regarding the effects of Lp(a) on platelet function remains inconclusive. Although Lp(a) and apo(a) may enhance platelet aggregation induced by arachidonic acid and thrombin receptor-activating peptide (TRAP), their effects vary depending on the agonist used and its concentration [21]. Conversely, Lp(a) has been reported to inhibit aggregation induced by low collagen concentrations and platelet-activating factor (PAF), partly through mechanisms independent of PAF-acetylhydrolase. [22,23]. These conflicting findings suggest that Lp(a) modulates platelet responses in a context-dependent manner, potentially influenced by interactions between apo(a), apoB-100/LDL, and platelet agonists. Lp(a) may modulate lipid metabolism and immune responses through several pathways. It is a major carrier of oxidized phospholipids, which promote endothelial dysfunction, lipid deposition and inflammatory signaling in atherosclerosis [24]. In addition, the apo(a) component of Lp(a) has been shown to interact with iC3b, a cleavage product of complement component C3. However, this association did not measurably affect C3 activation or degradation, and its functional significance remains unclear [25]. Collectively, these findings suggest that Lp(a) links lipid transport with inflammatory and immune processes, although the precise role of its interactions with the complement system requires further studies. The major molecular mechanisms underlying the role of Lp(a) in atherosclerosis and thrombosis are summarized in Figure 1. Higher concentrations of Lp(a) are associated with calcific aortic valve disease and more rapid hemodynamic progression of aortic stenosis [9,11]. Residual Lp(a)-associated risk persists during statin therapy [26], and very high concentrations may confer a lifetime risk comparable to that observed in heterozygous familial hypercholesterolemia [13,27]. Associations with atrial fibrillation and venous thromboembolism are less firmly established and should not be interpreted as equivalent to the evidence for ASCVD or aortic stenosis [18,28]. Similarly, the potential involvement of Lp(a) in cancer remains speculative. Experimental studies suggest that apo(a)-derived kringle fragments may inhibit angiogenesis and tumor growth, potentially through the modulation of endothelial signaling [29]. In addition, interference with plasminogen-dependent extracellular matrix degradation has been proposed as a mechanism that could limit tumor invasion and metastasis [30]. Conversely, the pro-inflammatory properties of Lp(a)-associated oxidized phospholipids raise the possibility of action on the tumor microenvironment [31]. However, these observations are largely based on experimental models and cannot be directly extrapolated to the biological activity of intact Lp(a) in human malignancies. Clinical studies have reported inconsistent associations between circulating Lp(a) concentrations and cancer risk, and a causal relationship has not been demonstrated.

### 2.2. Measurement and Interpretation

Lp(a) can be reported as particle concentration in nmol/L or mass concentration in mg/dL. Because apo(a) isoform size, and consequently the molecular mass of Lp(a), varies substantially among individuals, no single fixed conversion factor can accurately convert Lp(a) mass concentration (mg/dL) to molar concentration (nmol/L) across all samples. Results should therefore be reported in the units generated by the original assay, and fixed conversion or exact equivalence between thresholds expressed in mg/dL and nmol/L should be avoided. Assays with minimal sensitivity to apo(a) isoform size, calibrated using reference material traceable to the WHO/IFCC standard, are preferred; molar reporting in nmol/L is recommended where available [5,14].

**Figure 2:**
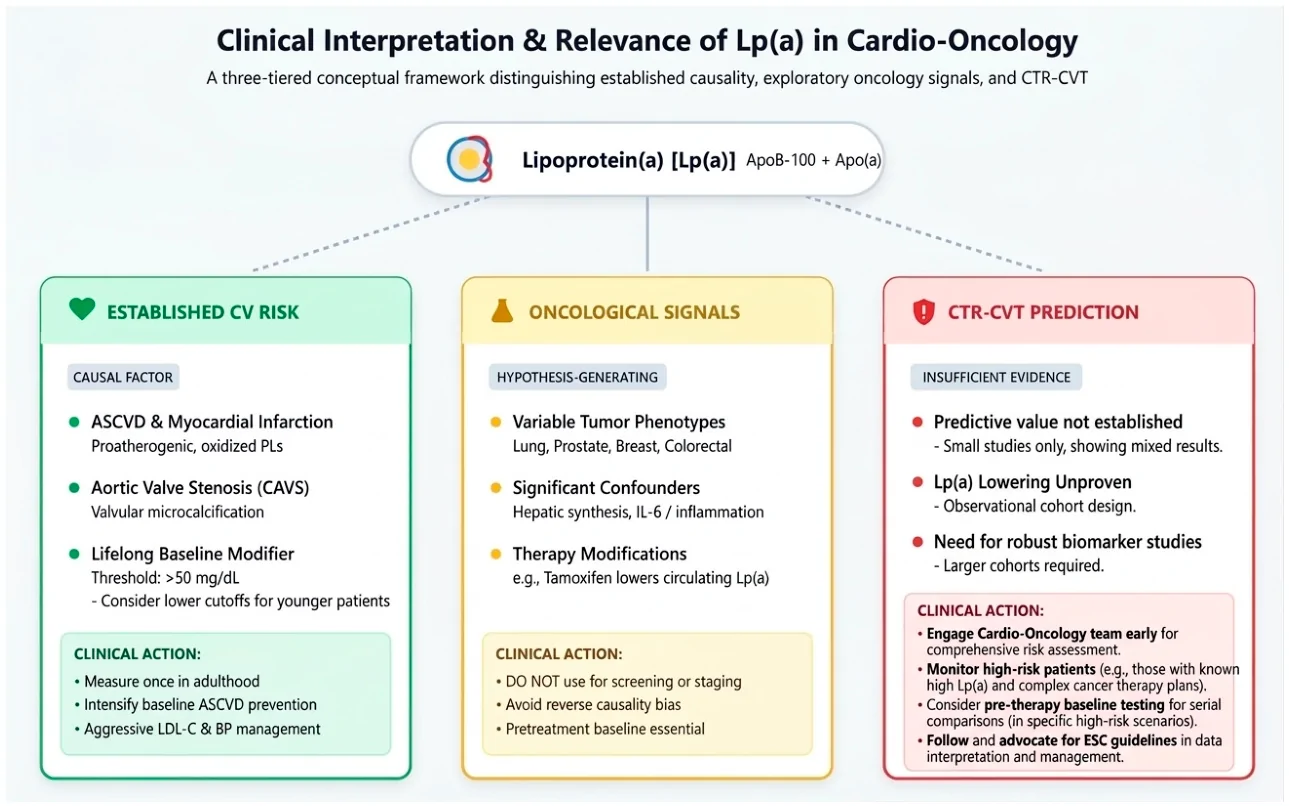
Clinical Interpretation and relevance of Lp(a) in Cardio-Oncology. Source: Created by the authors using Google’s Gemini image-generation tool, based on published scientific literature and verified by the authors.

Thresholds used to facilitate clinical interpretation differ among guidelines. The 2024 National Lipid Association focused update classifies Lp(a) concentrations below 75 nmol/L or 30 mg/dL as low risk, concentrations of 75–125 nmol/L or 30–50 mg/dL as intermediate risk, and concentrations of at least 125 nmol/L or 50 mg/dL as high risk [32]. The 2025 ESC/EAS focused update identifies Lp(a) concentrations above 50 mg/dL or 105 nmol/L as a cardiovascular risk modifier, whereas several North American publications use a threshold of 50 mg/dL or 125 nmol/L [13,14,33]. These values should be regarded as parallel clinical decision thresholds rather than exact conversions between mass and molar units. Importantly, Lp(a)-associated cardiovascular risk increases continuously across the concentration range; therefore, these categories should not be interpreted as discrete biological cut-offs [5]. Lp(a) concentrations should instead be interpreted within the patient’s overall cardiovascular risk profile, including established ASCVD, familial hypercholesterolemia, conventional risk factors, and exposure to potentially cardiotoxic anticancer therapies [Figure 2]. Population distributions also differ according to ancestry, with median Lp(a) concentrations generally being higher in Black populations and lower in East Asian populations than in populations of European ancestry. Nevertheless, elevated Lp(a) is associated with cardiovascular risk across ancestry groups, although the absolute risk associated with a given concentration may vary [5,34,35].

Several acquired conditions may modify Lp(a), including nephrotic syndrome, hypothyroidism, pregnancy, menopause, acute inflammatory states, and advanced liver disease [5,34]. This is particularly important in oncology. A result obtained during acute illness, marked systemic inflammation, severe hepatic dysfunction, or major treatment-related metabolic disturbance may not reflect the patient’s stable baseline. When the value is unexpected or potentially confounded, repeat measurement after clinical stabilization may be reasonable. ApoB must not be used as a surrogate for Lp(a): each Lp(a) particle contains apoB-100, but plasma apoB represents the total number of circulating apoB-containing particles and cannot determine the Lp(a) concentration.

## 3. Why Lp(a) May Matter in Cancer

Cancer and cardiovascular disease share risk factors and biological pathways, including chronic inflammation, oxidative stress, endothelial dysfunction, and thrombosis [1,2]. Anticancer therapies may additionally induce dyslipidemia, hypertension, insulin resistance, vascular injury, myocardial dysfunction, or thrombosis [36]. Against this background, elevated Lp(a) could plausibly amplify long-term ASCVD risk even when LDL cholesterol is well controlled. This hypothesis is clinically credible because the Lp(a)-associated component of risk is not eliminated by statins [26]. In patients who have undergone mediastinal radiotherapy the residual risk may be particularly important given the additional burden of vascular injury, which may accelerate atherosclerosis.

A study among 10,413 Japanese participants with no history of neoplasm at baseline, investigated whether low Lp(a) concentrations were associated with cancer mortality. In a median observation period of 4559 days, 316 patients died from cancer. The analysis has shown a positive correlation between low Lp(a) (lower than 80 mg/L) and increased cancer mortality [37]. On the other hand, the Copenhagen General Population Study on 109,440 participants found no significant association between low Lp(a) concentrations and cancer incidence (HR 1.06, 95% CI: 0.97–1.15) comparing the lowest quartile [2096 events] with the highest quartile [2018 events]; Lp(a) IQR: 4.7–29 mg/dL) [38].

The possibility that Lp(a) directly affects tumor biology is much less certain. Oxidized phospholipids, inflammatory signaling, fibrinolysis, and lipid availability could theoretically influence the tumor microenvironment, angiogenesis, immune responses, or metastatic behavior. Conversely, cancer may alter Lp(a) through hepatic synthesis, inflammation, cachexia, hormonal changes, or treatment. Cross-sectional differences in Lp(a) can therefore reflect reverse causation or confounding. Cancer-specific associations should be considered hypothesis-generating unless supported by prospective studies, with appropriate control of liver function, inflammatory status, treatment applied, and conventional lipid variables.

## 4. Lp(a), Cancer Phenotype, and Clinical Outcomes

### 4.1. Lung Cancer

Among observational cohort of untreated men with primary lung cancer, Lp(a) concentrations increased from stage I to stage III but decreased modestly in stage IV, suggesting a potentially non-linear association with disease stage [39]. The authors concluded, that additional studies with a larger number of patients were needed to confirm the association between Lp(a) and the presence and stage of lung cancer. They also mentioned core limitations, such as sex-restricted cohort, non-linear pattern and limited control of confounding. More recent research has investigated Lp(a) as a potential biomarker of treatment response and tumor phenotype. In a prospective single-center cohort of 227 Chinese patients with metastatic lung adenocarcinoma without actionable driver alterations and a programmed death-ligand 1 (PD-L1) tumor proportion score below 50% who received first-line chemoimmunotherapy, higher serum Lp(a) concentrations were independently associated with shorter progression-free survival (PFS) [40]. A model incorporating serum Lp(a), PD-L1 expression, and the presence of liver metastases demonstrated good discriminatory performance in randomly generated training and internal-validation subsets. In a separate exploratory analysis of a publicly available transcriptomic dataset, higher tumor LPA expression, so a parameter distinct from circulating Lp(a), was associated with lower expression of CD28 and lymphotoxin-α (LTA) and with enrichment of microenvironment [40]. Collectively, these findings suggest a possible relationship between tumor LPA-related biology and the local immune environment, but they neither establish causality nor demonstrate that circulating Lp(a) directly suppresses antitumor immunity.

A single-center retrospective study of 338 patients with advanced lung adenocarcinoma found that pretreatment Lp(a) concentrations above a data-derived threshold were associated with a lower prevalence of EGFR mutations. Among 123 patients with EGFR-mutated tumors treated with tyrosine kinase inhibitors, higher Lp(a) concentrations were independently associated with longer PFS (median, 16.1 vs. 9.6 months; HR, 0.42; 95% CI, 0.26–0.68) [41]. However, the cut-off of 20.48 mg/L was study-specific and data-derived and was substantially lower than thresholds commonly used for cardiovascular risk assessment. In another single-center retrospective study of 849 patients with resected NSCLC, preoperative Lp(a) concentrations were positively and linearly associated with baseline tumor diameter [42]. In the postoperative prognostic subset of 222 patients, higher Lp(a) was also associated with greater lung cancer mortality among patients aged ≥58 years (HR, 1.69), but not among younger patients [42]. Collectively, the inconsistent direction and subgroup dependence of the reported prognostic associations, together with the retrospective single-center designs, selected study populations, data-derived thresholds, small prognostic subsets, and lack of external validation, limit the current clinical applicability of Lp(a) as a biomarker in lung cancer.

### 4.2. Prostate Cancer

Early evidence provide limited insight into relationship between Lp(a) and prostate cancer. In a 1996 study primarily designed to evaluate the effects of androgen-deprivation therapy on lipid metabolism, baseline Lp(a) concentrations were compared in 50 men with prostate cancer treated with goserelin and 58 age-matched controls [43]. In addition, separate longitudinal analysis of 16 initially untreated patients was performed, who were assessed before and after three months of androgen-deprivation therapy with goserelin alone or in combination with flutamide. The androgen suppression had no effect on Lp(a) concentrations either in the cross-sectional or in the longitudinal analysis. It is yet to be established whether prostate cancer itself, independently of administered treatment, is associated with changes in Lp(a) levels.

In contrast, a single-center observational study of 376 patients with pathologically confirmed prostate cancer found higher median Lp(a) concentrations among patients with prostate-specific antigen (PSA) concentrations ≥100 ng/mL than among those with lower PSA concentrations [44]. In logistic regression analyses, patients in the highest Lp(a) quartile had greater odds of high-risk disease than those in the lowest quartile (unadjusted odds ratio [OR], 2.687; 95% confidence interval [CI], 1.113–6.491; *p* = 0.028; adjusted OR, 2.890; 95% CI, 1.148–7.274; *p* = 0.024). However, several limitations of this study should be acknowledged. The single-center design might restrict the applicability of these findings to broader populations. Furthermore, the observational nature of the study limited ability to draw conclusions regarding causality.

More recent findings have also been heterogeneous. A small case–control study published in 2026, comprising 35 men with metastatic prostate cancer, 35 with localized high-grade prostate cancer, and 35 controls at risk of prostate cancer, found no significant differences in Lp(a) or PCSK9 concentrations among the groups. By contrast, triglyceride, apolipoprotein C-III, ANGPTL3, and leptin concentrations were higher in patients with metastatic disease [45]. A multivariable Mendelian randomization analysis provided evidence of modest associations between genetically predicted Lp(a) concentrations and both overall prostate cancer risk (OR per standard-deviation increase, 1.068; 95% CI, 1.005–1.134; *p* = 0.034) and early-onset disease (OR, 1.150; 95% CI, 1.015–1.303; *p* = 0.028). The association with advanced prostate cancer was of borderline statistical significance (OR, 1.078; 95% CI, 0.999–1.163; *p* = 0.055) [46]. Although these genetic findings are less susceptible to conventional confounding and reverse causation, residual horizontal pleiotropy cannot be excluded, and the small effect estimates do not establish the clinical utility of Lp(a) as a prostate-cancer biomarker.

### 4.3. Breast and Ovarian Cancer

Studies investigating Lp(a) in breast cancer have yielded conflicting results. Several small studies reported higher Lp(a) concentrations in patients with breast cancer than in healthy controls [47,48]. In an early study comprising 18 patients with stage I disease, 21 with stage IV disease, and 22 healthy controls, Lp(a) concentrations were higher in both cancer groups than in controls and were also higher in patients with stage IV than in those with stage I disease [48]. A subsequent study reported elevated Lp(a) concentrations in patients with breast cancer and a decrease among patients who responded to chemotherapy [47].

In a Chinese study of 314 patients, Lp(a) concentrations were higher in patients with breast cancer than in healthy controls. Lp(a) was not significantly associated with estrogen- or progesterone-receptor status but was inversely associated with HER2 expression [49]. Nevertheless, the small sample size may affect the reliability and generalizability of the findings. Conversely, a study of 46 women found no significant differences in Lp(a) concentrations among those with benign breast disease, stage 0 breast cancer, and stage III breast cancer, and no association between Lp(a) and breast-disease severity after adjustment for age and body mass index [50].

These discrepancies may reflect small sample sizes, heterogeneous control groups, differences in ancestry, menopausal and metabolic status, treatment exposure or the timing of blood sampling, assay methodology, and tumor subtype. Overall, the available evidence is insufficient to support the use of Lp(a) as a diagnostic, prognostic, or treatment-response biomarker in breast cancer.

Evidence regarding Lp(a) in ovarian cancer is particularly limited. A historical study published in 1992 evaluated plasma apo(a)/Lp(a) and CA125 concentrations in healthy women and in patients with benign or malignant ovarian disease [51]. Apo(a)/Lp(a) concentrations did not provide clinically useful discrimination between women with and without ovarian cancer. Interpretation of these findings limited by the historical study design, age-stratified analyses, the use of an older assay reporting apo(a) concentrations in U/L, and the absence of contemporary assay standardization, multivariable adjustment, or external validation. Furthermore, the results have not been confirmed in modern prospective cohorts. Accordingly, current evidence is insufficient to support Lp(a) as a diagnostic, prognostic, or disease-monitoring biomarker in ovarian cancer.

### 4.4. Colorectal and Gastric Cancer

Lipid metabolism is extensively reprogrammed in colorectal cancer; however, this complex biology does not establish a specific role for Lp(a) [52]. A retrospective inpatient case–control study including 393 patients with colorectal cancer and 2429 controls reported a positive, approximately linear association between Lp(a) concentration and the odds of prevalent colorectal cancer [53]. In the prospective PRIME cohort, the highest Lp(a) category was also associated with a possible increase in colorectal cancer risk; however, the analysis included only 58 colorectal cancer cases, resulting in considerable statistical uncertainty [54]. Selection bias, reverse causation, the small number of prospective events, and residual confounding preclude causal interpretation and limit the clinical applicability of these findings.

In an observational study of 179 patients with stage III–IV gastric adenocarcinoma, peritoneal metastasis was associated with lower concentrations of total cholesterol, LDL-C, and apoB, as well as a lower apoB/apoA-I ratio [55]. In multivariable analysis, only lower total cholesterol remained independently associated with peritoneal metastasis. Although Lp(a) was measured, no significant association between Lp(a) concentration and peritoneal metastasis was reported. The observed lipid alterations should not be interpreted as evidence that lower lipid concentrations promote metastatic spread. Advanced disease, systemic inflammation, malnutrition, and cancer-associated cachexia may reduce circulating lipid concentrations and therefore provide an alternative, more likely explanation for these findings. Accordingly, this study should not be cited as evidence that Lp(a) is associated with peritoneal metastasis in gastric cancer.

### 4.5. Hepatocellular Carcinoma

Given that apolipoprotein(a) is synthesized primarily by hepatocytes, the lower Lp(a) concentrations observed in patients with hepatocellular carcinoma (HCC) may reflect reduced hepatic synthetic capacity rather than a cancer-specific protective or pathogenic mechanism [56,57]. Interestingly, one study reported higher Lp(a) concentrations in patients with HCC complicated by portal vein thrombosis than in those without portal vein thrombosis [58]. However, this finding does not establish that Lp(a) contributes causally to portal vein thrombosis, because hepatic functional reserve, cirrhosis severity, tumor burden, systemic inflammation, and the distinction between bland and tumor-associated portal vein thrombosis may all influence this association. Overall, the clinical interpretation of Lp(a) in HCC remains limited unless liver function, underlying liver disease, tumor stage, and thrombotic phenotype are carefully considered.

### 4.6. Pancreatic Cancer

In a retrospective single-center cohort of 364 patients with pathologically confirmed pancreatic cancer, higher baseline Lp(a) concentrations were associated with shorter overall survival (OS) and progression-free survival (PFS). After propensity score matching, median OS was 12.28 versus 27.67 months, and median PFS was 7.00 versus 11.30 months in the high- and low-Lp(a) groups, respectively [59]. In multivariable Cox regression analyses, higher Lp(a) remained independently associated with poorer OS (hazard ratio [HR], 2.11; 95% confidence interval [CI], 1.17–3.81) and PFS (HR, 2.14; 95% CI, 1.20–3.83). However, the Lp(a) cut-off of 29 mg/L was derived retrospectively using X-tile analysis and was substantially lower than thresholds commonly used for cardiovascular risk assessment. Despite the magnitude of the reported associations, evidence from a single retrospective cohort cannot determine whether Lp(a) has a causal role or instead represents an inflammatory or metabolic marker, a correlate of tumor biology, or a surrogate for unmeasured disease severity. External validation using prespecified and clinically interpretable Lp(a) thresholds is required.

Table 1 summarizes the principal clinical studies discussed in this review. Their heterogeneity in design, variations in treatment exposure, assay methods, threshold, study population and endpoints and timing of Lp(a) measurement precludes a unified estimate of association and limit comparability. These findings highlight the need for larger prospective studies with standardized methodologies.

## 5. Effects of Anticancer Therapy on Lp(a)

Anticancer therapies may alter lipid metabolism through hepatic, endocrine, inflammatory, and metabolic pathways [36]. Tamoxifen provides the clearest example of a treatment-associated change in Lp(a). A systematic review and meta-analysis of five studies involving 215 participants found that tamoxifen treatment was associated with a significant reduction in circulating Lp(a) concentrations (standardized mean difference, −0.41; 95% confidence interval, −0.68 to −0.14; *p* = 0.003) [61]. This effect is biologically consistent with the partial estrogen-agonist activity of tamoxifen in the liver. Reductions in Lp(a) have been reported both in patients with breast cancer and in healthy women [61,62]. In a placebo-controlled study of healthy women, mean Lp(a) concentrations decreased by 23% after one month of tamoxifen treatment, whereas they increased by 6% with placebo; no further substantial change was observed at two or six months [62]. Consequently, an Lp(a) measurement obtained after the initiation of tamoxifen may not accurately represent the pretreatment concentration.

Evidence regarding the effects of other anticancer therapies on Lp(a) is sparse. Although androgen-deprivation therapy, targeted therapies, immune checkpoint inhibitors, corticosteroids, and other cancer treatments may substantially alter conventional lipid parameters or affect hepatic, renal, endocrine, and inflammatory pathways, these effects cannot be assumed to translate into predictable changes in Lp(a). Treatment-specific longitudinal studies of Lp(a) remain limited. Studies designed to investigate such effects should obtain a pretreatment Lp(a) measurement, use standardized and preferably isoform-insensitive assays, and repeat measurements at predefined time points. Outside a research setting, repeat testing may be considered when the initial measurement was obtained during treatment or in the presence of a clinical condition likely to influence Lp(a) concentrations.

## 6. Lp(a), Cardiovascular Risk, and Cancer Therapy-Related Cardiovascular Toxicity

The established association of Lp(a) with ASCVD does not automatically qualify it as a biomarker of cancer therapy-related cardiovascular toxicity (CTR-CVT). CTR-CVT encompasses heterogeneous clinical phenotypes, including cardiac dysfunction and heart failure, myocarditis, vascular toxicity, myocardial ischemia, hypertension, arrhythmias, valvular disease, and thrombotic events [3]. A biomarker associated with one pathophysiological mechanism cannot be assumed to predict all these outcomes.

In a prospective cohort of 2192 patients with newly diagnosed or recurrent malignancies, Kraler et al. reported 50 major adverse cardiovascular events (MACE) during a median follow-up of 23.4 months. Baseline Lp(a) concentrations were not significantly associated with MACE (adjusted subdistribution hazard ratio per doubling of Lp(a): 1.10, 95% CI 0.89–1.36), with no cardiovascular death treated as a competing event [60]. Notably, the study focused on MACE rather than the broader spectrum of CTR-CVT, limiting the applicability of its findings to other cardiotoxicity phenotypes. These results do not support the use of Lp(a) as a validated general predictor of MACE or cancer therapy-related cardiotoxicity in an unselected cancer population. However, they do not exclude its potential value in selected patients exposed to therapies that promote atherosclerosis or in cancer survivors with a long life expectancy. Studies stratified by treatment class and based on prospectively adjudicated cardiovascular phenotypes are required.

The current clinical role of Lp(a) in cardio-oncology is therefore more limited and better established. Rather than being a biomarker for various CTR-CVT phenotypes such as heart failure, myocarditis, or arrhythmias, Lp(a) measurement identifies an inherited predisposition to ASCVD and calcific aortic valve disease that coexists with cancer and may become more clinically relevant when anticancer treatment adds vascular, inflammatory, or metabolic stress.

Lp(a) should be interpreted as part of comprehensive baseline cardiovascular risk assessment and should complement, rather than replace, treatment-specific surveillance, cardiac imaging, electrocardiography, cardiac troponin and natriuretic peptide measurements, and assessment of conventional cardiovascular risk factors when these evaluations are clinically indicated [3]. At present, there is insufficient evidence to recommend Lp(a) as a stand-alone biomarker for predicting CTR-CVT. Although Lp(a) is an established marker of ASCVD risk, its clinical utility in patients with cancer remains poorly defined. In particular, it is unclear whether cancer modifies the association between Lp(a) levels and cardiovascular outcomes, to what extent Lp(a) improves the predictive accuracy of existing cardio-oncology models, and whether its measurement translates into meaningful alterations in clinical management.

## 7. Assessment of Lp(a) in Cardio-Oncology Practice

Lp(a) measurement may be incorporated into comprehensive cardiovascular risk assessment in cardio-oncology. Its potential clinical implications are summarized in Table 2; however, the categories presented in the table represent a narrative appraisal rather than a formal evidence-grading system.

Measurement of Lp(a) at least once in adulthood is reasonable as part of lifetime cardiovascular risk assessment, particularly when no previous reliable result is available [13,14]. Testing may be especially informative in patients with premature ASCVD or a family history of premature ASCVD, familial hypercholesterolemia, calcific aortic valve disease, or an unexpectedly high burden of atherosclerosis [13,14]. In patients with cancer, the resulting information may complement—rather than replace—the treatment-specific baseline cardiovascular toxicity risk assessment recommended by the 2022 ESC cardio-oncology guidelines [3]. At present, Lp(a) should be interpreted primarily as an established cardiovascular risk modifier rather than as a cancer-specific or cancer therapy-related cardiovascular toxicity biomarker.

When Lp(a) concentration is elevated, a prompt refinement of global cardiovascular risk should be performed, rather than cancer-specific surveillance. Management should emphasize intensive control of LDL cholesterol and other modifiable risk factors, smoking cessation, blood-pressure and glycemic control, physical activity as tolerated, and optimization of preventive therapy according to the patient’s overall risk, prognosis, treatment plan, bleeding risk, drug interactions, and preferences. Statins remain indicated when otherwise appropriate, even though they do not lower Lp(a) and may modestly increase it [26,63].

Notably, elevated Lp(a) alone should not trigger additional echocardiography, cardiac magnetic resonance imaging, or biomarker monitoring beyond what is recommended for the relevant anticancer regimen and baseline cardiovascular risk. Likewise, there is no evidence that serial Lp(a) testing improves detection of cardiotoxicity. Repeat measurement may be considered when the initial value was obtained during a potentially confounding clinical state or after a therapy known or suspected to alter Lp(a).

## 8. Current and Emerging Lp(a)-Lowering Therapies

Lifestyle measures remain essential for reducing total cardiovascular risk but they have little or no effect on the genetically determined Lp(a) concentration. Statins do not lower Lp(a); however, they should not be withheld when indicated, given the well-established LDL-cholesterol-lowering benefits [26,63].

PCSK9 inhibitors generally reduce Lp(a) by approximately 20–30% in addition to substantial LDL-cholesterol lowering, although they are not approved specifically as Lp(a)-targeted therapy [64]. Lipoprotein apheresis results in significant, acute reductions in Lp(a) levels and remains a therapeutic option in selected regions for patients with very high Lp(a) and progressive ASCVD despite optimal treatment [65]; however, its use is limited by availability, cost, and the need for repeated procedures [66].

RNA-targeted agents reduce hepatic apo(a) synthesis. Small interfering RNA agents, including olpasiran, zerlasiran, and lepodisiran, have produced substantial and durable Lp(a) reductions in early- and mid-phase studies [67,68,69]. Pelacarsen is an antisense oligonucleotide that targets LPA messenger RNA [70,71]. Oral small molecules such as muvalaplin aim to inhibit assembly of the Lp(a) particle. These approaches demonstrate pharmacological target engagement, but reduction in a biomarker is not equivalent to improvement in clinical outcomes.

This distinction became especially important in September 2026. In company-reported topline results from the phase III Lp(a)HORIZON trial (Novartis), pelacarsen lowered Lp(a) but did not meet the primary endpoint of reducing a composite of cardiovascular death, non-fatal myocardial infarction, non-fatal stroke, and urgent coronary revascularization in the overall study population [72]. However, these findings derive from a corporate press release, and full peer-reviewed results and prespecified subgroup analyses are still awaited for complete interpretation. At present, these findings reinforce the need to distinguish among Lp(a) as a causal risk factor, Lp(a) lowering as a pharmacodynamic effect, and a specific intervention’s ability to improve outcomes. No evidence supports Lp(a)-directed treatment for cancer-related outcomes.

## 9. Knowledge Gaps and Research Priorities

The evidence base has several recurring limitations: small samples; retrospective or cross-sectional designs; heterogeneous assays and thresholds; inconsistent reporting of units; inadequate adjustment for ancestry, liver and renal function, inflammation, nutritional status, and treatment; and selective evaluation of tumor types and outcomes. Publication bias is also plausible. These limitations make it difficult to determine whether Lp(a) is a causal contributor, a host-risk marker, or a consequence of cancer and its treatment. Therefore, it is yet impossible to give a definitive answers to the questions raised at the beginning. Moment in the patient history when Lp(a) should be measured is not well defined, and studies presented different approaches (1). Available research suggests associations between Lp(a) and cancer occurrence or prognosis. However, evidence is limited and inconsistent, therefore this issue requires further research (2). Lp(a) seems to be more important for assessing risk of long-term ASCVD cardiotoxicity events rather than acute cases occurring during active cancer treatment. It is though hard to say whether this risk is greater than in the general population (3).

Future studies should:

1. Enroll adequately powered, prospective cardio-oncology cohorts with pretreatment Lp(a) measurement;
2. Use isoform-insensitive assays and report original units without fixed conversion between mg/dL and nmol/L;
3. Prespecify tumor type, stage, treatment exposure, cardiovascular phenotype, and follow-up interval;
4. Adjust for ancestry, menopausal and hormonal status, hepatic and renal function, inflammation, nutritional state, and conventional cardiovascular risk factors;
5. Distinguish ASCVD events from myocardial dysfunction, myocarditis, arrhythmia, hypertension, venous thromboembolism, and other CTR-CVT phenotypes;
6. Investigate mechanisms using paired clinical samples and experimental models before attributing immune or metastatic effects directly to Lp(a).

Dedicated analyses are also needed to determine whether Lp(a) modifies cardiovascular risk from therapies with vascular or metabolic effects and whether cancer survivors with high Lp(a) derive differential benefit from established LDL-lowering strategies. Trials of Lp(a)-targeted agents should report cancer history and treatment exposure when feasible, but oncology-specific use should not be inferred from general cardiovascular trials.

## 10. Conclusions

The aim of this review is to sum up the evidence on Lp(a) and cancer with its potential implications for cardiovascular risk assessment in cardio-oncology. While, Lp(a)’s importance in ASCVD is established, the current role of Lp(a) in cardio-oncology is limited. Rather than being a biomarker for various CTR-CVT phenotypes such as heart failure, myocarditis, or arrhythmias, high Lp(a) identifies an inherited predisposition to ASCVD and calcific aortic valve disease that coexists with cancer and may become more clinically relevant in patients exposed to treatment-induced vascular toxicity, in whom Lp(a) measurement should prompt strict cardiovascular surveillance. In this context, Lp(a) should be incorporated into comprehensive cardiovascular risk evaluation, considering pre-existing cardiac diseases, conventional risk factors, malignancy-related characteristics, and the vascular effects of anticancer treatment. As an additional risk modifier rather than a marker of toxicity, Lp(a) may contribute to the optimization of preventive measures, including management of modifiable risk factors, guideline-directed lipid-lowering therapy, and proper follow-up. Associations between Lp(a) levels and cancer occurrence, phenotype, treatment response, or prognosis have been reported across several malignancies, but are heterogeneous and largely observational. They do not justify using Lp(a) for cancer screening, staging, prognostication, or treatment selection. Evidence that Lp(a) independently predicts ASCVD events of CTR-CVT is insufficient, and the largest prospective mixed-cancer biomarker study did not demonstrate an independent association with MACE.

Addressing the questions raised in the Introduction, first, the optimal time of Lp(a) measurement in cancer patients remains uncertain. Second, although several studies have reported associations between Lp(a) concentrations and cancer occurrence, the evidence remains scarce to implement it as an oncological biomarker. Third, Lp(a) may be more relevant for long-term ASCVD risk assessment in cancer survivors, than for predicting acute complications during treatment.

Carefully designed prospective and mechanistic studies are required to establish whether Lp(a) provides incremental value in cardio-oncology and whether any Lp(a)-directed intervention benefits patients with cancer.

## Figures and Tables

**Table 1:** Clinical studies evaluating Lp(a) in patients with cancer.

Cancer Type	Study and Design	Population and Follow-Up Duration	Lp(a) Assessment (Timing, Thresholds) and Main Findings	Main Adjustment Variables	Adjusted Effect Estimates (95% CI)	Lp(a) Assay Details and Units	Principal Limitation
Lung	Yang et al.; observational cohort [39]	418 untreated male patients with lung cancer (stages I–IV) and 65 healthy male controls. No longitudinal follow-up.	Fasting serum samples collected before anticancer treatment. Median Lp(a): 157 mg/L in patients vs. 110 mg/L in controls (*p* = 0.004). Lp(a) concentrations increased progressively from stages I to III, with a slight decrease in stage IV	Age, HDL-C, CEA, hypertension, and diabetes.	Age-adjusted OR 1.022 (1.001–1.044); multivariable OR 1.039 (1.006–1.073) *p* = 0.040. Lost significance after adjustment for smoking.	Nephelometry (Olympus AU 2700 analyzer; (Olympus Medical Engineering Company, Tokyo, Japan) Leadman reagents (Sino-German Beijing Leadman Biotech. Ltd., Beijing, China); mg/L.	Sex-restricted cohort; non-linear pattern; limited control of confounding
Lung	Li et al.; Prospective cohort with internal validation [40]	227 patients with metastatic lung cancer receiving first-line chemoimmunotherapy Median follow-up: 11.36 months.	Baseline Lp(a) with median of 20.0 mg/dL, analyzed as a continuous variable, with no predefined cutoff. In a multivariable prognostic model, higher Lp(a) concentrations, analysed as a continuous variable, were associated with shorter PFS	PD-L1 expression (TPS category) and liver metastasis	Multivariable Cox regression: HR 1.025 (95% CI 1.016–1.033; *p* < 0.001) per 1 mg/dL increase in Lp(a) for PFS	Latex-enhanced immunoturbidimetric assay. Manufacturer and analyzer: NR; mg/dL	Single-country cohort; exploratory transcriptomics; no external validation
Lung	Liu et al.; retrospective single-center study [41]	338 patients with stage IIIB–IV lung adenocarcinoma; 123 EGFR-mutant patients receiving EGFR-TKIs included in the PFS analysis. Median follow-up: 31.8 months (PFS cohort).	Lp(a) measured within 1 month before therapy. ROC-derived cutoff: 20.48 mg/L (high >20.48; low ≤20.48). EGFR mutation rate: 48.0% vs. 65.5% (high vs. low Lp(a), *p* = 0.001). Median PFS: 16.1 vs. 9.6 months (*p* = 0.015). Higher Lp(a) was inversely associated with EGFR mutation prevalence in the overall cohort, while among EGFR-mutant patients receiving EGFR-TKIs, higher Lp(a) was associated with longer PFS.	EGFR mutation model: sex, age, smoking, chronic pulmonary disease, brain and bone metastases. PFS model: sex, age, smoking, EGFR genotype, TNM stage, bone metastases, and EGFR-TKI generation.	Multivariable logistic regression (EGFR mutation): adjusted OR 0.41 (95% CI 0.25–0.66; *p* < 0.001). Multivariable Cox regression (PFS): adjusted HR 0.42 (95% CI 0.26–0.68; *p* < 0.001). Both estimates compare high vs. low Lp(a) (>20.48 vs. ≤20.48 mg/L).	NR assay method and manufacturer; mg/L.	Retrospective design and data-derived threshold
NSCLC	Liu et al.; retrospective study [42]	849 patients undergoing radical NSCLC resection; 222 included in the postoperative survival analysis (105 aged <58 years; 117 aged ≥58 years). Follow-up up to 5 years.	Lp(a) was positively correlated with baseline tumor diameter (r = 0.10; *p* for linear trend = 0.002). Among patients aged ≥58 years, higher Lp(a) was associated with increased mortality in unadjusted and partially adjusted analyses, but not in the fully adjusted model.	Model 1: sex, BMI, alcohol consumption, smoking. Model 2: additionally adjusted for aCCI, neoadjuvant treatment, histological subtype, and baseline tumor diameter.	Patients aged ≥58 years (high vs. low Lp(a)): Unadjusted: HR 1.69 (*p* = 0.026). Partially adjusted (Model 1): HR 1.63 (95% CI 1.01–2.63; *p* = 0.044). Fully adjusted (Model 2): association no longer statistically significant.	NR assay method, manufacturer; mg/L.	Retrospective design; uncertain clinical utility
Prostate	Wang and Zhang; observational cohort [44]	376 patients with newly diagnosed, prostate cancer (54 low-/intermediate-risk; 322 high-risk). No longitudinal follow-up.	Lp(a) measured before surgery, androgen deprivation or other therapies. Higher Lp(a) concentrations were associated with high-risk clinicopathological features (median 224 vs. 165 mg/L; *p* = 0.022) and PSA ≥100 ng/mL (*p* = 0.002).	Age, BMI, hypertension, diabetes, coronary artery disease, and lipid-lowering medication use.	Multivariable logistic regression: adjusted OR 2.890 (95% CI 1.148–7.274; *p* = 0.024) for high-risk prostate cancer, comparing the highest (Q4: >381 mg/L) vs. lowest (Q1: <104 mg/L) Lp(a) quartile.	Turbidimetric immunoassay (LASAY Lp(a) auto; SHIMA Laboratories Co., Ltd, Itabashi-ku, Tokyo, Japan); mg/L.	Single-center observational study; association with phenotype, not incident cancer
Prostate	Ioannidou et al.; multivariable Mendelian randomization [46]	79,166 prostate cancer cases and 61,106 controls; European ancestry. No longitudinal follow-up.	Genetically predicted Lp(a), analyzed per 1-SD increase in inverse rank-normalized levels; no clinical concentration cutoff. Higher Lp(a) levels were associated with a modestly increased risk of overall and early-onset prostate cancer in a Mendelian randomization analysis.	Multivariable MR adjusted for genetically predicted HDL-C, LDL-C, and triglycerides; additional analyses included BMI, AST, ALT, and creatinine.	Multivariable MR per 1-SD increase in genetically predicted Lp(a): overall PCa, OR 1.068 (1.005–1.134); advanced PCa, OR 1.078 (0.999–1.163); early-onset PCa, OR 1.150 (1.015–1.303).	No assay-specific measurements or concentration thresholds used in the MR models. Genetically predicted Lp(a) received from UK Biobank.	Instrument and ancestry limitations; small effect estimates
Breast	Wong Chong et al.; cross-sectional study [50]	46 women: 23 with stage III breast cancer and 23 age-matched controls. No longitudinal follow-up.	Lp(a) measured before initiation of cancer therapy, no predefined cutoff. Median Lp(a): 35 nmol/L (IQR 12–169) in stage III breast cancer vs. 42 nmol/L (IQR 13–136) in controls (*p* = 0.51). No association with breast disease Severity.	Age and BMI (partial Spearman correlation analysis); case–control groups were age-matched.	Age- and BMI-adjusted partial Spearman correlation: ρ = −0.043; *p* = 0.781. Adjusted OR/HR and 95% CI: NR.	Roche Modular analytical platform; specific Lp(a) assay methodology not reported; nmol/L	Small sample size
Breast	Xu et al.; observational study [49]	550 participants: 314 breast cancer patients, 51 patients with benign breast tumors, and 185 healthy controls. No longitudinal follow-up.	Lp(a) measured before cancer treatment; no defined cutoff. Median Lp(a): 163.95 ng/mL in breast cancer, 153.60 ng/mL in benign tumors, and 112.90 ng/mL in healthy controls. Higher Lp(a) were in breast cancer compared to healthy controls (*p* = 0.00019) and were inversely associated with HER2 expression, with no significant associations observed for ER or PR status.	Age adjustment was applied to analyse clinicopathological characteristics, not to the primary cancer–control comparison.	Age-adjusted logistic regression (Model 2; HER2+++ reference): HER2-negative, OR 1.002 (95% CI 1.000–1.004; *p* = 0.018); HER2++, OR 1.003 (95% CI 1.001–1.005; *p* = 0.016). No adjusted effect estimate reported for breast cancer vs. healthy controls.	Immunoturbidimetric assay (Meikang Biotech Co., Ltd., Shanghai, China); Beckman Coulter AU5800 analyzer; Brea, USA; ng/mL.	Cross-sectional design and residual confounding
Colorectal	Wang et al.; retrospective inpatient study [53]	2822 inpatients undergoing colonoscopy: 393 newly diagnosed cancer cases and 2429 controls with normal colonoscopy. No longitudinal follow-up.	Lp(a) measured during hospitalization. Median Lp(a): 198 mg/L (IQR 100–384) in CRC vs. 138 mg/L (IQR 77–290) in controls (*p* < 0.001). Higher Lp(a) concentrations were independently associated with greater odds of colorectal cancer (*p* = 0.002), with a positive linear relationship observed in adjusted analyses.	Age, sex, weight, marital status, family history of CRC, alcohol consumption, smoking, albumin, ALT, β2-microglobulin, HDL-C, total cholesterol, hypertension, and diabetes mellitus.	Fully adjusted logistic regression (Model III): OR 1.08 (95% CI 1.03–1.13; *p* = 0.002) per 100 mg/L increase in Lp(a).	Assay methodology, manufacturer, and analyzer not reported; mg/L	Selection bias; temporality and causality cannot be established
Multiple cancers	PRIME; prospective cohort [54]	5196 cancer-free men aged 50–59 years; 456 incident cancers (126 prostate, 60 lung, 58 colorectal). Median follow-up: 10 years.	Lp(a) measured before cancer diagnosis. Quartiles: Q1 <10; Q2 10–<55; Q3 55–<170; Q4 ≥170 mg/L. No statistically significant association with overall or site-specific cancer incidence. The highest Lp(a) levels demonstrated a nonsignificant trend toward increased incidence of colorectal cancer, differing associations observed across sites.	Primary Cox models: age, study center, smoking status, and alcohol consumption. Additional adjustment for education, employment status, BMI, systolic BP, physical activity, dyslipidemia, and lipid-lowering therapy was explored.	Multivariable Cox regression (Q4 vs. Q1): all-site cancer, HR 1.29 (95% CI 0.99–1.67); lung cancer, HR 2.00 (0.94–4.29); colorectal cancer, HR 1.63 (0.79–3.35); prostate cancer, HR 0.84 (0.49–1.45). All associations were nonsignificant.	Immunoenzymatic assay; central laboratory (Institut Pasteur de Lille, France); Manufacturer not reported; detection limit 10 mg/L	Few site-specific events
Gastric	Zhang et al.; observational study [55]	179 patients with stage III–IV gastric adenocarcinoma (23 stage III, 156 stage IV); 125 men and 54 women. No longitudinal follow-up.	Lp(a) measured initially; median 219.60 mg/L (IQR 136.10–346.30). No predefined cutoff. Lp(a) was not significantly associated with peritoneal, lung, ovarian, liver, bone, or distant lymph node metastases.	Multivariable logistic models included selected clinical and lipid variables, but Lp(a) was not retained.	No adjusted effect estimates for Lp(a). Univariate logistic regression: peritoneal metastasis, OR 1.00 (95% CI 0.99–1.01); lung metastasis, OR 1.01 (1.00–1.02); ovarian metastasis, OR 1.01 (0.97–1.05); all *p* > 0.05.	Assay methodology and manufacturer not reported; mg/L.	Assay characteristics and major clinical confounders of Lp(a) insufficiently addressed.
HCC	Malaguarnera et al.; observational study [58]	144 participants: 44 patients with HCC and portal vein thrombosis (PVT), 50 patients with HCC without PVT, and 50 healthy controls; aged 65–80 years. No longitudinal follow-up.	No predefined Lp(a) cutoff. Mean Lp(a) concentrations were 102.1 ± 12.9 mg/dL in HCC with PVT, 64.8 ± 12.8 mg/dL in HCC without PVT, and 27.1 ± 12.1 mg/dL in healthy controls. Lp(a) was significantly higher in HCC with PVT than in HCC without PVT (*p* < 0.001) and healthy controls (*p* < 0.001).	No clearly specified multivariable adjustment for the association between Lp(a) and PVT or HCC.	No adjusted effect estimates or 95% CIs reported. The authors reported OR 9.21 for PVT and OR 6.33 for HCC, without specifying the Lp(a) contrast, adjustment variables, or confidence intervals.	Commercial enzyme-linked immunosorbent assay (ELISA; Immuno GmbH, Heidelberg, Germany); mg/dL.	Small observational study; tumor versus bland thrombosis may be difficult to distinguish
Pancreatic	Liu et al.; retrospective single-center cohort [59]	364 patients with pancreatic cancer (215 underwent radical surgery); 98 patients included in the matched overall cohort. Median follow-up: 13.55 months.	Baseline serum Lp(a) assessed before anticancer treatment. Study-specific, data-derived baseline Lp(a) of ≥29 mg/L, was associated with shorter OS and PFS in patients with pancreatic cancer. After PSM, median OS was 12.28 vs. 27.67 months (*p* = 0.003), and median PFS was 7.00 vs. 11.30 months (*p* = 0.002), for high vs. low Lp(a), respectively.	Age, sex, BMI, tumor location, TNM stage, surgical status, and serum lipid profiles. Multivariable Cox models additionally included surgical status, nodal and metastatic status, triglycerides, and CEA, sex, and triglycerides (PFS model).	Post-PSM multivariable Cox regression (Lp(a) ≥29 vs. <29 mg/L): OS: aHR 2.11 (95% CI 1.17–3.81; *p* = 0.013). PFS: aHR 2.14 (95% CI 1.20–3.83; *p* = 0.010).	Latex-enhanced immunoturbidimetric assay; analyzers and reagents supplied by Medicalsystem Biotechnology; Ningbo, China); mg/L.	Retrospective design; single center; potential systemic-disease confounding
Mixed malignancies	Kraler et al.; prospective biomarker cohort [60]	2192 cancer patients; median follow-up 23.4 months; 50 MACE	Lp(a) measured before initiation of anticancer therapy and analyzed as a continuous variable. No significant association between Lp(a) and 2-year MACE was observed. MACE incidence was 3.0% (95% CI 1.6–5.1%) for Lp(a) ≥ 75th percentile vs. 2.3% (1.5–3.3%) below the 75th percentile. Absolute Lp(a) concentration units and percentile cutoff were not specified in the main article.	Age, sex, smoking, established atherosclerotic cardiovascular disease (ASCVD), and cancer type.	Multivariable Fine–Gray competing-risk regression: adjusted subdistribution hazard ratio 1.10 (95% CI 0.89–1.36) per doubling of Lp(a) concentration for 2-year MACE.	Roche Tina-quant Lp(a) assay, Cobas c702 platform (Roche Diagnostics; Rotkreuz, Switzerland). Measurement units not specified.	Heterogeneous cancers and treatments; not designed for individual cardiotoxic regimens

Abbreviations: aHR, adjusted Hazard Ratio; aCCI, age-adjusted Charlson Comorbidity Index; CRC, Colorectal Cancer; EGFR, epidermal growth factor receptor; ER, estrogen receptor; GWAS, genome-wide association study; HCC, hepatocellular carcinoma; HER2, human epidermal growth factor receptor 2; Lp(a), lipoprotein(a); MACE, major adverse cardiovascular events; NSCLC, non-small-cell lung cancer; OD; odds ration; OS, overall survival; PCa, prostate cancer; PFS, progression-free survival; PR, progesterone receptor; UK, united kingdom, NR, not reported; NA, not applicable; CI, confidence interval; HDL-C, high-density lipoprotein cholesterol; CEA, carcinoembryonic antigen; TPS, tumor proportion score; HR, hazard ratio; Multivariable MR, multivariable Mendelian randomization.

**Table 2:** Potential clinical implications of Lp(a) measurement in cardio-oncology.

Clinical Question	Current Evidence	Practical Interpretation
Does Lp(a) refine lifetime ASCVD risk in a patient with cancer?	Established in general cardiovascular populations; biologically applicable to oncology	Use as a risk modifier within comprehensive cardiovascular assessment
Does elevated Lp(a) identify patients who need more intensive management of modifiable risk factors?	Indirect but clinically compelling	Optimize LDL-C, blood pressure, diabetes, smoking status, weight, and physical activity according to total risk
Does Lp(a) predict cancer occurrence, stage, or prognosis?	Emerging and inconsistent observational evidence	Do not use for cancer screening, staging, treatment selection, or prognostication outside research
Does Lp(a) predict CTR-CVT?	Insufficient evidence for CTR-CVT; the largest prospective mixed-cancer cohort found no significant association between Lp(a) and MACE but did not assess the broader spectrum of CTR-CVT.	Lp(a) should not be used as a stand-alone predictor of cardiotoxicity or to determine surveillance intensity.
Should Lp(a) be measured repeatedly during cancer treatment?	No evidence for routine serial testing	Usually a one-time measurement; repeat only when a reversible confounder or treatment effect is suspected
Should elevated Lp(a) lead to Lp(a)-specific drug therapy?	No approved targeted therapy with proven outcome benefit at the time of writing	Treat overall cardiovascular risk; consider specialist referral or clinical trials in selected patients

Abbreviations: ASCVD, atherosclerotic cardiovascular disease; CTR-CVT, cancer therapy-related cardiovascular toxicity; LDL-C, low-density lipoprotein cholesterol; Lp(a), lipoprotein(a).

## Data Availability

No new data were created or analyzed in this study. Data sharing is not applicable to this article.
